# Supplementary material for: Resolving the full spectrum of human genome variation using Linked-Reads
Source: Genome Res. 2019 Apr;29(4):635–45. doi: 10.1101/gr.234443.118 (PMC6442396; doi:10.1101/gr.234443.118)
Supplement: Supplemental Material [file supp_gr.234443.118_Supplemental_longranger-2.2.zip › longranger-2.2.tar.bz2!/lr2.2-public/lib/stan/cmdstan/stan/doc/api/html/namespacemembers_e.html]

Stan: Namespace Members


|  |  |
| --- | --- |
|  | Stan  2.5.0  probability, sampling & optimization |


- Main Page
- Related Pages
- Namespaces
- Classes
- Files

- Namespace List
- Namespace Members

- All
- Functions
- Variables
- Typedefs
- Enumerations
- Enumerator

- a
- b
- c
- d
- e
- f
- g
- h
- i
- j
- l
- m
- n
- o
- p
- q
- r
- s
- t
- u
- v
- w


All Classes Namespaces Files Functions Variables Typedefs Enumerations Enumerator Friends Macros Pages

Here is a list of all namespace members with links to the namespace documentation for each member:

### - e -

- E
  : stan::math
- e()
  : stan::math
- eigenvalues\_sym()
  : stan::math
- eigenvectors\_sym()
  : stan::math
- elt\_divide()
  : stan::math
- elt\_division\_f
  : stan::gm
- elt\_multiplication\_f
  : stan::gm
- elt\_multiply()
  : stan::math
- EMPTY\_EXP\_VECTOR()
  : stan::gm
- empty\_nested()
  : stan::agrad
- ends\_with()
  : stan::gm
- EOL()
  : stan::gm
- EOL2()
  : stan::gm
- EPSILON
  : stan::math
- erf()
  : stan::agrad
- erfc()
  : stan::agrad
- exp()
  : stan::agrad
  , stan::math
- exp2()
  : stan::agrad
  , stan::math
- exp\_mod\_normal\_ccdf\_log()
  : stan::prob
- exp\_mod\_normal\_cdf()
  : stan::prob
- exp\_mod\_normal\_cdf\_log()
  : stan::prob
- exp\_mod\_normal\_log()
  : stan::prob
- exp\_mod\_normal\_rng()
  : stan::prob
- expm1()
  : stan::agrad
- exponential\_ccdf\_log()
  : stan::prob
- exponential\_cdf()
  : stan::prob
- exponential\_cdf\_log()
  : stan::prob
- exponential\_log()
  : stan::prob
- exponential\_rng()
  : stan::prob
- exponentiation\_f
  : stan::gm
- expression\_as\_statement\_f
  : stan::gm


---

[ Stan Home Page ]

*© 2011–2014,
Stan Development Team.*
